# Caring Across Cultures: A Cross-Sectional Study of Cultural Competence Among Nurses in Norway

**DOI:** 10.1177/10436596261423159

**Published:** 2026-02-23

**Authors:** Tariq Alkhaled, Berit Johannessen, Birgit Lie, Lise-Merete Alpers, Gudrun Rohde

**Affiliations:** 1 University of Agder, Kristiansand, Norway; 2 Sorlandet Hospital, Kristiansand, Norway; 3 VID Specialized University, Oslo, Norway

**Keywords:** cultural competence, demographic factors, health care, nurses, regional variations, self-efficacy, training programs

## Abstract

**Introduction:**

Cultural competence is essential for equitable, safe, and person-centered care in diverse populations. Nurses need to develop cultural understanding and abilities to provide culturally competent care. This study aimed to describe cultural competence among nurses working in somatic hospitals and investigate associations between demographic characteristics, knowledge and experience, self-efficacy, and cultural competence of nurses.

**Method:**

An online survey on nurses’ cultural competence was conducted with 201 nurses from three hospitals in Norway. Logistic regression analyses were used to examine associations between demographic characteristics, knowledge, and self-efficacy with cultural competence.

**Results:**

Significant associations were identified between age, gender, birthplace, international experience, and hospital affiliation with dimensions of cultural competence. Knowledge and self-efficacy of nurses were identified as critical factors positively associated with cultural competence.

**Discussion:**

Demographic factors influence nurses’ cultural competence and underline the importance of tailored training. Promoting knowledge and self-efficacy can improve culturally congruent care.

## Introduction

Global migration is steadily increasing, with approximately 281 million international migrants residing worldwide (United Nations, 2020). In Norway, the migrant population comprised 21.4% of the total population in 2025 (Statistics Norway, 2025), and this proportion is expected to grow, further increasing cultural diversity within health care facilities. This demographic shift underscores the relevance of cultural competence among nurses to provide equitable and effective care for patients from diverse cultural backgrounds (Kaihlanen et al., 2019; Tosun et al., 2021; World Health Organization, 2023).

Cultural competence is defined as “an ongoing process in which the health care professional continuously strives to achieve the ability and availability to work effectively within the cultural context of the patient (individual, family, community)” (Campinha-Bacote, 2002, p. 181). This involves integrating cultural desire, awareness, knowledge, skills, and encounters into clinical practice.

In this study, knowledge refers to nurses’ factual understanding of cultural norms, values, and health-related practices (e.g., religious dietary rules, death rituals). Cultural competence, in contrast, encompasses the integration of this knowledge with attitudes, communication skills, and behaviors that enable effective care delivery to patients from diverse backgrounds (Leininger & McFarland, 2002; Theodosopoulos et al., 2024). Thus, knowledge represents a component of cultural competence but does not, on its own, ensure competent practice.

Lack of cultural competence may result in negative consequences for both nurses and patients. For nurses, it can lead to moral distress and ethical tension, for example, when family visitation or culturally based care requests conflict with hospital policies, increased job strain, frustration, and burnout. In addition, it can affect the planning of nursing care, lead to prejudice and discrimination, result in misinterpretation of patients’ needs, and lead to incorrect diagnoses and treatment (Antón-Solanas et al., 2021).

For patients, inadequate culturally competent care can result in barriers to health care access, communication challenges, and implicit bias (Berlinger & Berlinger, 2017; Dobrowolska et al., 2020). In contrast, culturally competent nurses positively affect the quality of nursing care, patient satisfaction, and patient outcomes (Cai et al., 2021; Tang et al., 2019). Nevertheless, delivering culturally competent care can be challenging in clinical practice, particularly in complex health care settings (Anzini et al., 2025; Handtke et al., 2019).

In addition to knowledge, attitudes, and communication skills, nurses’ self-efficacy may play an important role in shaping their cultural competence. Studies have shown that higher self-efficacy is associated with greater cultural competence among nurses (Ham & Tak, 2022; Han & Jeong, 2023). Therefore, self-efficacy can be considered a relevant factor contributing to culturally competent practice.

The immigrant population is increasing in Norway, but few studies have been conducted to investigate the cultural competence of health care workers. This study was conducted to describe cultural competence among nurses working in somatic hospitals and investigate potential associations between demographic characteristics, knowledge and experience, self-efficacy, and cultural competence of nurses.

## Method

### Ethics Approval and Consent to Participate

Participants were informed about the study objectives through an online questionnaire and provided electronic informed consent before completing the survey. We ensured the confidentiality and anonymity of the collected data. Ethical approval was obtained from the University of Agder and the Norwegian Agency for Shared Services in Education and Research.

### Design

We conducted a cross-sectional study using an online survey that was available to be completed between April and August 2023.

### Setting and Participants

Three hospitals were selected in different regions of Norway—northern, southern, and central Norway—to represent hospitals across the country. The hospitals served diverse demographic areas, influencing nurses’ exposure to cultural variations and competence (Statistics Norway, 2025).

The inclusion criteria required a minimum of 1 year of work experience in hospitals. Nurses with less than 1 year of experience and those in temporary positions were excluded. Invitations, containing a link to the online questionnaire, were sent to the head nurses in various departments. They subsequently distributed the invitations to the nurses within their respective departments via email. All nurses employed at the three somatic hospitals (i.e., general medical and surgical hospitals), totaling 1783, were invited to participate. Of the 265 responses received, 201 participants completed the entire survey.

### Survey Instruments

We selected action-focused items based on clinical practice because we aimed to measure observable, practice-related aspects of cultural competence that directly affect patient care and are actionable for training and quality improvement (Alpers & Hanssen, 2014). The survey, incorporating a combination of close-ended and open-ended questions, was employed to collect variables based on the questionnaire previously developed and used by (Alpers, 2017). The questionnaire consists of six sets of items, including Likert-type scale questions.

The development of this comprehensive questionnaire, which included a mix of question types, was validated and supported by insights from an earlier published study (Alpers & Hanssen, 2014). A total of 25 statements (Additional file 1) were crafted to measure cultural competence across five domains: (a) illness, health behavior, and pain; (b) collaboration, attitudes, and behavior; (c) communication and collaboration with interpreters; (d) death and dying; and (e) culture, religion, and dietary considerations. For each statement, participants had six response options: 1–2: Strongly agree/agree, 3: Neither agree nor disagree, 4–5: Disagree/strongly disagree, and 6: I don’t know. In addition, there were four open-ended questions.

The self-efficacy scale, using the Norwegian version of the generalized self-efficacy scale (GSE), was incorporated into the survey to assess the optimistic self-beliefs of individuals in coping with the demands, tasks, and challenges of life (Bonsaksen et al., 2019). The scale, consisting of 10 items measured on a 4-point Likert-type scale (1: *completely wrong* to 4: *completely right*), demonstrated high internal consistency with a Cronbach’s α coefficient of .879 in the present study. The scores provided by the participants for each item were summed and divided by 10 to generate a GSE score. Higher scores on this scale corresponded to higher levels of generalized self-efficacy. A previous study has demonstrated the reliability and validity of the GSE (Luszczynska et al., 2005).

The survey we employed comprised a section about the demographic, educational, and professional background of the nurses and had a total of 25 items about cultural competence. Five items from the 25 were selected based on expert review and face validity as binary outcome variables of subsequent logistic regression (Bursac et al., 2008).

The dependent variables derived from our survey data pertained to the following aspects: (a) illness, health behavior, and pain (“Assessing pain levels in patients from an ethnic minority background can be challenging due to their different expressions of pain compared with Norwegian patients”); (b) collaboration, attitudes, and behavior (“I always try to comply with the wishes of patients/relatives from an ethnic minority background”); (c) communication and collaboration with interpreters (“It is difficult to communicate well with patients through an interpreter”); (d) death and dying (“I have sufficient knowledge of deathbed rituals when a patient from a minority background passes away and needs to be cared for”); and (e) culture, religion, and dietary considerations (“I have sufficient knowledge of religious dietary rules”).

All these dependent variables were represented in the form of disagreement (by grouping “don’t know,” “strongly disagree,” “disagree,” and “neither agree nor disagree”) and agreement (by grouping “strongly agree” and “agree”). The list of independent variables included gender, age groups of participants, place of birth, languages other than Norwegian, hospital, years of experience, education other than nursing, resided or studied in another country, knowledge regarding ethnic minority patients, and the total score of self-efficacy. The independent variables were selected based on associations revealed in previous studies and clinical experience (Bursac et al., 2008).

### Statistical Analysis

Data analysis was performed using Statistical Package for the Social Sciences (SPSS) version 29. The data file was created in SPSS following the predefined coding manual for labeling responses of the participants and then preprocessed to remove missing and outlier observations (Meyers et al., 2013). Descriptive data are presented for all collected variables.

Continuous data are described using the median and range or the mean and *SD*, depending on variable distribution. Categorical data are presented as counts and percentages. Binary logistic regression was employed to investigate the associations of experience-related variables, knowledge-related variables, self-efficacy, and demographic variables with dependent variables (illness, health behavior, and pain; collaboration, attitudes, and behavior; communication and collaboration with interpreters; death and dying; and culture, religion, and dietary considerations).

Finally, a logistic regression model was fitted using a backward conditional variable selection approach on the dependent variables (illness, health behavior, and pain; collaboration, attitudes, and behavior; communication and collaboration with interpreters; death and dying; and culture, religion, and dietary considerations). A backward conditional selection approach was chosen because it starts with all potential predictors and stepwise removes those that do not contribute significantly to the model. This approach allowed us to identify the most meaningful predictors and produce a more parsimonious and interpretable model (Lu & Wang, 2024).

The goodness-of-fit indices were computed to assess the model adequacy. The results are reported as odds ratios (ORs) with 95% confidence intervals, and the *R*^2^ was assessed by Nagelkerke *R*^2^. In addition, *p*-values < .05 were considered statistically significant.

## Results

Table 1 presents the demographic characteristics of the study population (*n* = 201), consisting primarily of female nurses (89.1%) born in Scandinavia (88.5%) and working in three hospitals located in South (48.8%), North (36.3%), and Central Norway (14.9%). The age distribution was fairly evenly spread, with the largest group aged 22 to 30 years (35.8%). The nursing experience was varied, with 25.4% and 22.9% having <3 and >21 years of experience, respectively.

**Table 1.** Demographic Characteristics of the Study Population (n = 201).

Variable	*N*	%
Gender		
Male	22	10.9
Female	179	89.1
Age groups (years)		
22–30	72	35.8
31–40	58	28.9
41–50	36	17.9
>51	35	17.4
Place of birth		
Scandinavia	178	88.5
Europe, outside Scandinavia	11	5.5
America	4	2.0
Asia	6	3.0
Africa	2	1.0
Hospitals		
Southern Norway	89	48.8
Northern Norway	73	36.3
Central Norway	30	14.9
Experience groups (years)		
<3	51	25.4
3–5	29	14.4
6–10	34	16.9
11–15	28	13.9
16–20	13	6.5
>21	46	22.9
Proficiency in other languages		
Yes	188	93.5
No	13	6.5
Other degrees in addition to nursing qualification		
Yes	94	46.8
No	107	53.2
Resided or studied in another country		
Yes	68	33.8
No	133	66.2
Knowledge regarding patients from an ethnic minority background		
Yes	74	36.8
No	127	63.2

Most participants had proficiency in other languages, mainly English (93.5%), and 46.8% held more than a bachelor’s degree. One-third of the nurses had experience residing or studying in another country (33.8%), while 36.8% reported that they had knowledge regarding patients from an ethnic minority background.

### Associations of Cultural Competence of Nurses Using Binary Logistic Regression

To examine the association of various demographic and experience-related factors with the cultural competence of nurses in Norway, we implemented a binary logistic regression model and backward conditional selection to identify which variables were independently and significantly associated with each cultural competence domain outcome. The results of the fitted models are presented in Table 2.

**Table 2.** Logistic Regression Analysis and Final Backward Model for Associations of Cultural Competence and Patient Interaction Among Nurses.

	Assessing pain levels in patients from an ethnic minority can be challenging				I always try to comply with the wishes of patients/relatives from an ethnic background				It is difficult to communicate effectively with patients through an interpreter	
	Initial model		Backward selection		Initial model		Backward selection		Initial model	
Variable	OR [95% CI]	*P*-value	OR [95% CI]	*P-*value	OR [95% CI]	*P*-value	OR [95% CI]	*P*-value	OR [95% CI]	*P*-value
Gender										
Female Ref-	1.45 [0.50, 4.23])	.500			0.33 [0.09, 1.24]	.101			1.07 [0.36, 3.22]	.903
Age group (years)										
22–30 Ref-		.411				.666				.170
31–40	0.85 [0.32, 2.25]	.748			0.61 [0.16, 2.26]	.460			1.03 [0.36, 2.92]	.962
41–50	2.11 [0.50, 8.98]	.312			0.56 [0.09, 3.50]	.537			0.47 [0.10, 2.11]	.321
>51	3.89 [0.60, 25.27]	.155			1.21 [0.12, 12.45]	.876			0.13 [0.02, .95]	.**044**
Place of birth										
Scandinavia Ref-		.276				.780				.**004**
Europe, outside Scandinavia	0.68 [0.17, 2.69]	.585			2.13 [0.22, 20.22]	.512			0.13 [0.03, .58]	.**007**
Outside Europe	3.07 [0.65, 14.54]	.158			.86 [0.15, 4.85]	.863			0.13 [0.03, .60]	.**009**
Languages other than Norwegian										
No Ref-	0.85 [0.22, 3.26]	.813			.22 [0.02, 2.20]	.195			0.88 [0.21, 3.70]	.863
Hospital										
Southern Norway Ref-		<.**001**		**0.002**		.**020**		.**034**		.964
Northern Norway	0.25 [0.12, 0.52]	<.**001**	0.31 [0.16, 0.59]	<.**001**	4.81 [1.56, 14.79]	.**006**	3.93 [1.38, 11.20]	.**010**	0.92 [0.43, 1.97]	.838
Central Norway	0.66 [0.25, 1.75]	.403	0.66 [0.28, 1.59]	.354	1.01 [0.31, 3.23]	.999	1.11 [0.39, 3.12]	.845	0.89 [0.31, 2.52]	.822
Years of experience										
<3 Ref-		.367				.914				.593
3–5	1.36 [0.47, 3.90]	.571			0.85 [0.21, 3.48]	.826			0.74 [0.22, 2.47]	.628
6–10	0.2.55 [0.79, 8.24]	.118			1.73 [0.34, 8.85]	.513			0.45 [0.14, 1.43]	.175
11–15	0.83 [0.23, 2.97]	0.775			0.81 [0.16, 4.06]	.793			0.93 [0.22, 3.95]	.920
16–20	0.48 [0.09, 2.65]	.401			0.95 [0.10, 9.05]	.965			1.33 [0.21, 8.59]	.765
>21	0.42 [0.07, 2.42]	.331			0.55 [0.07, 4.65]	.582			2.16 [0.33, 14.39]	.425
Education other than nursing										
No Ref-	0.84 [0.42, 1.69]	.633			0.87 [0.34, 2.20]	.770			1.38 [0.66, 2.89]	.397
Resided or studied in another country										
No Ref-	0.59 [0.27, 1.29]	.185			0.98 [0.36, 2.62]	.962			3.66 [1.39, 9.61]	.**009**
Knowledge regarding ethnic minority patients										
No Ref-	0.50 [0.25, 1.03]	.060			1.46 [0.55, 3.92]	.448			.71 [0.34, 1.47]	.354
The total score of self-efficacy	1.09 [0.99, 1.20]	.081			1.13 [0.99, 1.29]	.079			1.03 [0.91, 1.11]	.948
Explained variance	19.6%		12.2%		16.6%		8.0%		17.0%	
	It is difficult to communicate effectively with patients through an interpreter		I have sufficient knowledge of deathbed rituals				I have sufficient knowledge of religious dietary rules			
	Backward selection		Initial model		Backward selection		Initial model		Backward selection	
Variable	OR [95% CI]	*P*-value	OR [95% CI]	*P*-value	OR [95% CI]	*P*-value	OR [95% CI]	*P*-value	OR [95% CI]	*P*-value
Gender										
Female Ref-			1.13 [0.07, 19.40]	.936			3.86 [1.17, 12.70]	.**026**	3.48 [1.13, 10.71]	.**030**
Age group (years)										
22–30 Ref-				.685				.635		
31–40			0.13 [0.00, 4.34]	.251			1.50 [0.52, 4.33]	.454		
41–50			0.15 [0.00, 6.15]	.316			0.62 [0.13, 2.91]	.543		
>51			0.00 [0.00, .]	.997			0.60 [0.08, 4.45]	.621		
Place of birth										
Scandinavia Ref-		.**003**		.290				.259		
Europe, outside Scandinavia	0.14 [0.04, .57]	.**006**	0.10 [0.00, 7.13]	.287			2.10 [0.44, 9.93]	.351		
Outside Europe	0.17 [0.04, .65]	.**009**	3.67 [0.11, 127.29]	.472			3.30 [0.67, 16.34]	.144		
Languages other than Norwegian										
No Ref-			0.15 [0.01, 6.85]	.327			2.47 [0.59, 10.45]	.218		
Hospital										
Southern Norway Ref-				.**009**		.**003**		.**001**		<.**001**
Northern Norway			2.98 [0.25, 35.36]	.387	3.01 [0.41, 21.84]	.276	0.28 [0.11, 0.68]	.**005**	0.29 [0.13, 0.65]	.**003**
Central Norway			131.99 [5.06, 3441.36]	.**003**	32.50 [3.98, 265.43]	.**001**	2.37 [0.86, 6.53]	.095	2.04 [0.78, 5.33]	.145
Years of experience										
<3 Ref-				.607				.657		
3–5			0.00 [0.00]	.997			0.75 [0.23, 2.43]	.629		
6–10			0.29 [0.02, 4.04]	.360			0.46 [0.13, 1.66]	.239		
11–15			8.39 [0.24, 294.22]	.241			0.74 [0.18, 3.15]	.688		
16–20			13.015 [0.213, 796.388]	.222			2.485 [0.365, 16.896]	.352		
>21			0.00 [0.00]	.997			1.57 [0.24, 10.15]	.636		
Education other than nursing										
No Ref-			1.85 [0.31, 11.09]	.500			0.72 [0.32, 1.63]	.435		
Resided or studied in another country										
No Ref-	3.23 [1.35, 7.73]	.**008**	0.49 [0.04, 5.97]	.578			1.16 [0.50, 2.72]	.725		
Knowledge regarding ethnic minority patients										
No Ref-			18.14 [1.29, 255.71]	.**032**	9.14 [1.66, 50.52]	**0.011**	6.74 [3.07, 14.79]	<.**001**	6.05 [2.97, 12.30]	<.**001**
The total score of self-efficacy			0.88 [0.69, 1.14]	.331			1.07 [0.97, 1.19]	.189		
Explained variance	10.0%		54.5%		47.8%		39.5%		35.6%	

Bold values in Table 2 indicate statistically significant results (p < .05).

#### Competence to Map Pain

A significant association was found between hospital affiliation and reporting difficulties in assessing pain among ethnic minority patients (*p* < .001). Nurses affiliated with Northern Norway were approximately 75% less likely to report difficulties in assessing pain (OR = 0.25, 95% CI: [0.12, 0.52]) compared with those affiliated with Southern Norway.

In the backward selection, hospital affiliation was the only variable found to be statistically significant in Southern (*p* = .002), Northern (OR = 0.31, 95% CI: [0.16, 0.59], *p* < .001), and Central Norway (OR = 0.66, 95% CI: [0.28, 1.59], *p* = .354).

#### Cooperation as Compliance With the Wishes of the Patients

Hospital affiliation had a significant association with the cooperation of nurses with patients and their relatives (*p* = .02). Nurses affiliated with the Northern Norway hospital had a significantly higher likelihood of cooperating with patients and their relatives (OR = 4.81, 95% CI: [1.56, 14.79], *p* = .006) compared with those affiliated with the Southern Norway hospital.

In the backward selection, hospital affiliation was the only variable found to be statistically significant in Southern (*p* = .034), Northern (OR = 3.93, 95% CI: [1.38, 11.20], *p* = .01), and Central Norway (OR = 1.11, 95% CI: [0.39, 3.12], *p* = .845).

#### Communication and Use of an Interpreter

Nurses aged > 51 years showed a significant association with reporting difficulties in communication with patients through an interpreter (OR = 0.13, 95% CI: [0.02, 0.95], *p* = .044). In addition, the place of birth had a significant association with reporting difficulties in communication with patients through an interpreter (*p* = .004). In particular, nurses who were born in Europe but outside of Scandinavia (OR = 0.13, 95% CI: [0.31, 0.58], *p* = .007) and those born outside of Europe (OR = 0.13, 95% CI: [0.03, 0.60], *p* = .009) were more likely to report difficulties in communicating with the patients through an interpreter.

Having experience from another country had a significant association with the use of an interpreter. Nurses who had resided or studied in another country (OR = 3.66, 95% CI: [1.39, 9.61], *p* = .009) were 3.6 times more likely to report difficulties in communication with patients through an interpreter.

In the backward selection, the variables for place of birth in Scandinavia (*p* = .003), in Europe but outside Scandinavia (OR = 0.14, 95% CI: [0.04, 0.57], *p* = .006), and outside Europe (OR = 0.17, 95% CI: [0.04, 0.65], *p* = .009) and resided or studied in another country (OR = 3.23, 95% CI: [1.35, 7.73], *p* = .008) were found to be statistically significant.

#### Awareness About the Deathbed Experiences

The variable “Affiliation with a hospital” had a significant association with awareness about the deathbed experiences (*p* = .009). Nurses affiliated with the Central Norway hospital had a significantly higher likelihood of reporting awareness of deathbed experiences than those affiliated with the Southern Norway hospital (OR = 131.99, 95% CI: [5.06, 3441.36], *p* = .003). In addition, a significant association was found between the self-reported knowledge of nurses regarding patients from ethnic minority backgrounds (OR = 18.14, 95% CI: [1.29, 255.71], *p =* .032) and their agreement with their understanding of rituals associated with the end-of-life care for these patients.

In the backward selection, the variables for hospital affiliation in Southern (*p* = .003), Northern (OR = 3.01, 95% CI: [0.41, 21.84], *p* = .28), and Central Norway (OR = 32.50, 95% CI: [3.98, 265.43], *p* = .001) and the self-reported knowledge of nurses regarding patients from ethnic minority backgrounds (OR = 9.14, 95% CI: [1.65, 50.52], *p* = .011) were found to be statistically significant.

#### Awareness of Religious Dietary Rules

The gender of the nurses had a significant association with the awareness of religious dietary rules (*p* = .026). Female nurses were nearly four times more likely to report sufficient understanding of religious dietary rules than male nurses (OR = 3.86, 95% CI: [1.17, 12.70]). In addition, hospital affiliation showed a significant association with awareness of religious dietary rules (*p* = .001). Nurses affiliated with the Northern Norway hospital (OR = 0.28, 95% CI: [0.11, 0.68], *p* = .005) were less likely to agree that they have sufficient knowledge of religious dietary rules than those affiliated with the Southern Norway hospital. Nurses who reported a sufficient level of knowledge regarding patients from ethnic minority backgrounds also reported higher awareness of religious dietary rules (OR = 6.74, 95% CI: [3.07, 14.79], *p* < .001).

In the backward selection, the variables for gender (OR = 3.48, 95% CI: [1.13, 10.71], *p* = .03), hospital affiliation in Southern (*p* < .001), Northern (OR = 0.29, 95% CI: [0.13, 0.65], *p* = .003), and Central Norway (OR = 2.04, 95% CI: [0.78, 5.33], *p* = .145) and the self-reported knowledge of nurses regarding patients from ethnic minority backgrounds (OR = 6.05, 95% CI: [2.97, 12.30], *p* < .001) were found to be statistically significant.

## Discussion

The study findings revealed a significant association of age, gender, hospital affiliation, international experience, and the self-reported knowledge of nurses regarding patients from ethnic minority backgrounds with the cultural competence of nurses. Moreover, there was a trend for higher score-self-efficacy scores to be associated with higher cultural competence; however, the results did not reach the level of statistical significance. The findings will be further discussed, emphasizing demographic variables, international experience, and the interaction between knowledge, self-efficacy, and cultural competence.

### Demographic Variables and Cultural Competence

The gender of the nurse was significantly associated with their cultural competence in terms of knowledge of religious dietary rules. Female nurses were nearly four times more likely to report sufficient understanding of religious diet than male nurses. This finding aligns with previous findings suggesting that female health care providers may have a heightened sensitivity to dietary considerations because of their traditionally central roles in family nutrition (Sawari et al., 2018).

Age demonstrated a statistically significant association with the reporting of difficulties in communication with patients through an interpreter. Nurses aged > 51 years were more likely to face difficulties in effectively communicating with patients through an interpreter. This finding may reflect greater awareness of perceived communication challenges, rather than lower competence. This aligns with previous findings showing that cultural competence was positively associated with age and years of experience (Almutairi et al., 2017; Birhanu et al., 2023; Bunjitpimol et al., 2016; Urbanavičė et al., 2025).

The present study revealed a substantial association between hospital affiliation and nurses’ self-reported cultural competence. In general, nurses affiliated with the Northern Norway hospital reported higher cultural competence than those affiliated with Central and Southern Norway hospitals. This finding may be attributed to the diverse patient population in Northern Norway, where a large focus on cultural differences is necessary because of the presence of a large Sami population in the area (Statistics Norway, 2022). This prompted nurses to develop enhanced skills in culturally sensitive pain assessment, cooperation with patients, and awareness of religious dietary rules (Birhanu et al., 2023; Cai et al., 2021; Kaihlanen et al., 2019).

Regarding the awareness of deathbed rituals among nurses based on hospital affiliation, nurses from Central Norway reported a higher likelihood of awareness of deathbed rituals. Similar to hospitals with a diverse patient population, dedicated training programs focused on cultural competence tend to have a positive effect on the nursing staff (Douglas et al., 2014; Kaihlanen et al., 2019). This finding is consistent with previous findings highlighting the importance of institutional support in enhancing cultural competence (Butler et al., 2020; Soleimani & Yarahmadi, 2023; Weech-Maldonado et al., 2011).

### Contextual Factors: Birthplace and International Experiences

The place of birth and international experiences of nurses can affect cultural competence (Almutairi et al., 2017; Cai et al., 2021). The present study found that nurses born in Europe but outside of Scandinavia, as well as those born outside of Europe, were more likely to report communication difficulties when using interpreters. Communication difficulties may arise because Norwegian is the second language for both these nurses and the interpreters. The existing literature explains the difficulties of nurses in communicating with patients through variables, such as dialectal variations, cultural sensitivity, cultural competence, adaptability, the impact of cultural backgrounds, and potential differences in educational and training backgrounds (Alhamami, 2022; Ali & Watson, 2018; Dobrowolska et al., 2020; Soleimani & Yarahmadi, 2023).

A significant association existed between international experience (having resided or studied in another country) and communication difficulties. Nurses with international exposure were more likely to express such concerns. This finding might suggest that exposure to different cultural and linguistic contexts may contribute to heightened cultural competence. These findings align with previous studies demonstrating that nurses exposed to other cultures increased their cultural competence, enabling them to effectively engage with a diverse patient population (Hovland et al., 2021; Urbanavičė et al., 2025).

### The Interplay of Knowledge and Experience, Self-Efficacy, and Cultural Competence

The level of knowledge regarding patients from ethnic minority backgrounds was significantly associated with cultural competence. Nurses with a higher level of knowledge regarding patients from ethnic minority backgrounds were more likely to show an understanding of religious dietary rules and have sufficient knowledge of deathbed rituals. This association can be explained by factors such as a comprehensive educational background, exposure to cultural competence training, clinical experiences with diverse patient populations, institutional support, ongoing self-assessment, a commitment to patient-centered care, and individual efforts for continuous professional development (Hovland & Johannessen, 2019; Lin et al., 2015).

Collectively, these elements contribute to nurses with greater knowledge reporting higher cultural competence in understanding and addressing specific cultural aspects of patient care (Tosun et al., 2021). Consistent with previous findings, the knowledge of nurses regarding ethnic minority patients showed a strong positive association with all facets of cultural competence (Vella et al., 2022; Zeleke et al., 2024).

Similarly, self-efficacy had a positive effect on cultural competence, particularly in the cooperation of nurses with patients and relatives from ethnic minority backgrounds. This finding suggests that nurses who feel confident in their abilities are more likely to engage effectively with patients from diverse cultural backgrounds, and it is consistent with previous findings emphasizing the importance of self-efficacy in cultural competence training (Ham & Tak, 2022; Han & Jeong, 2023; Pirhofer et al., 2022).

### Implications for Practice and Education

Our findings might have an important role in nursing practice and education. Developing training initiatives that focus on cultural competence and providing institutional support for continuous education, cultural competence training, and ongoing professional development are needed. Cultivating a supportive environment for nurses to enhance their cultural competence, encouraging diversity within health care teams to expose nurses to various cultural perspectives, and fostering a culture of continuous improvement by encouraging nurses to regularly self-assess their cultural competence are important. Demonstrating cultural competence is a complex process that requires alignment between contextual factors, such as institutional support, and individual factors, including knowledge, confidence, and experience.

### Strengths and Limitations

The study has some limitations. First, the online survey produced a relatively low response rate (12%), which raises concerns about potential response bias and limits the representativeness of the findings. However, the anonymity of the online survey may have helped to reduce social desirability bias (Joinson, 1999). Another limitation of the study is the low percentage of male participants, which was only 11%. In addition, the questionnaire used was only partially validated and primarily descriptive in nature (Alpers & Hanssen, 2014). This limitation may affect the generalizability and precision of the findings.

One notable strength of this study is the intentional inclusion of hospitals from different regions, enhancing the relevance of the findings across diverse geographic contexts. Another strength of this study is its exploration of various dimensions of cultural competence among nurses, providing a nuanced understanding of the concept in clinical practice.

## Conclusion

This study identified several significant associations between demographic and contextual factors and nurses’ cultural competence. Hospital affiliation was associated with various domains of cultural competence, highlighting regional variations. Female nurses reported higher awareness of religious dietary rules, while older nurses and nurses with international experience reported greater difficulties in interpreter-mediated communication. In addition, nurses who reported higher levels of knowledge regarding ethnic minority patients demonstrated significantly greater awareness of religious dietary practices and deathbed rituals. Self-efficacy was positively associated with cultural competence across several domains.

These findings emphasize the importance of addressing cultural competence as both an individual and organizational responsibility. Future efforts should focus on enhancing nurses’ knowledge, self-efficacy, and interpreter-related communication skills through structured education and institutional support. Further research is needed to assess targeted interventions and to explore how organizational contexts influence culturally competent nursing care.

## Supplemental Material

sj-docx-1-tcn-10.1177_10436596261423159 – Supplemental material for Caring Across Cultures: A Cross-Sectional Study of Cultural Competence Among Nurses in Norway Supplemental material, sj-docx-1-tcn-10.1177_10436596261423159 for Caring Across Cultures: A Cross-Sectional Study of Cultural Competence Among Nurses in Norway by Tariq Alkhaled, Berit Johannessen, Birgit Lie, Lise-Merete Alpers and Gudrun Rohde in Journal of Transcultural Nursing
