# Supplementary material for: Caring Across Cultures: A Cross-Sectional Study of Cultural Competence Among Nurses in Norway
Source: J Transcult Nurs. 2026 Feb 23;37(4):675–84. doi: 10.1177/10436596261423159 (PMC13276121; doi:10.1177/10436596261423159)
Supplement: sj-docx-1-tcn-10.1177_10436596261423159 – Supplemental material for Caring Across Cultures: A Cross-Sectional Study of Cultural Competence Among Nurses in Norway [file sj-docx-1-tcn-10.1177_10436596261423159.docx]

**Supplementary Table 1:** Response of nurses to cultural competence instrument item

| ***Items*** | ***Strongly disagree*** | | ***Disagree*** | | ***Neither agree nor disagree*** | | ***Agree*** | | ***Strongly agree*** | | ***I don’t know*** | |
| --- | --- | --- | --- | --- | --- | --- | --- | --- | --- | --- | --- | --- |
|  | **N** | **%** | **N** | **%** | **N** | **%** | **N** | **%** | **N** | **%** | **N** | **%** |
| **Experience and knowledge** | | | | | | | | | | | | |
| We have many patients from an ethnic minority background in my department. | 0 | 0.0 | 8 | 4.0 | 54 | 26.9 | 118 | 58.7 | 17 | 8.5 | 4 | 2.0 |
| I have extensive experience with patients from ethnic minority backgrounds. | 5 | 2.5 | 41 | 20.4 | 78 | 38.8 | 68 | 33.8 | 9 | 4.5 | 0 | 0.0 |
| Working with patients from an ethnic minority background is different than working with ethnically Norwegian patients. | 0 | 0.0 | 6 | 3.0 | 34 | 16.9 | 134 | 66.7 | 23 | 11.4 | 4 | 2.0 |
| I am often uncertain when dealing with patients and/or relatives from an ethnic minority background. | 7 | 3.5 | 66 | 32.8 | 82 | 40.8 | 40 | 19.9 | 6 | 3.0 | 0 | 0.0 |
| If I have questions about patients from an ethnic minority background, there is no one to ask. | 6 | 3.0 | 73 | 36.3 | 63 | 31.3 | 40 | 19.9 | 9 | 4.5 | 10 | 5.0 |
| There is an internship opportunity for dealing with patients from diverse cultural or religious backgrounds. | 64 | 31.8 | 87 | 43.3 | 23 | 11.4 | 9 | 4.5 | 2 | 1.0 | 16 | 8.0 |
| I possess sufficient knowledge regarding patients from an ethnic minority background to provide these patients with quality care and nursing. | 3 | 1.5 | 38 | 18.9 | 83 | 41.3 | 65 | 32.3 | 9 | 4.5 | 3 | 1.5 |
| I prefer to avoid working with patients from ethnic minority backgrounds. | 96 | 47.8 | 81 | 40.3 | 21 | 10.4 | 2 | 1.0 | 1 | 0.5 | 0 | 0.0 |
| I am committed to not discriminating between patients and treating everyone equally, regardless of their ethnic or religious background. | 0 | 0.0 | 8 | 4.0 | 12 | 6.0 | 73 | 36.3 | 107 | 53.2 | 1 | 0.5 |
| I never contact my superior when I lack competence in relation to working with patients/relatives from an ethnic minority background. | 25 | 12.4 | 88 | 43.8 | 42 | 20.9 | 26 | 12.9 | 12 | 6.0 | 8 | 4.0 |
| **Illness, health behavior, and pain** | | | | | | | | | | | | |
| I possess knowledge of alternative perspectives on disease and treatment beyond the conventional “Norwegian” biomedical approach. | 2 | 1.0 | 13 | 6.5 | 69 | 34.3 | 89 | 44-3 | 11 | 5.5 | 17 | 8.5 |
| Assessing pain levels in patients from an ethnic minority background can be challenging due to their distinct expressions of pain compared with those of Norwegian patients. | 6 | 3.0 | 11 | 5.5 | 51 | 25.4 | 98 | 48.8 | 25 | 12.4 | 10 | 5.0 |
| **Cooperation with patients and relatives** | | | | | | | | | | | | |
| Engaging with patients/relatives from an ethnic minority background is stimulating. | 0 | 0.0 | 4 | 2.0 | 55 | 27.4 | 110 | 54.7 | 31 | 15.4 | 1 | 0.5 |
| It is educational to work with ethnic minority patients. | 0 | 0.0 | 1 | 0.5 | 35 | 17.4 | 126 | 62.7 | 39 | 19.4 | 0 | 0.0 |
| Patients from an ethnic minority background should manage their pain instead of making noise when they are in pain. | 90 | 44.8 | 70 | 34.8 | 25 | 12.4 | 7 | 3.5 | 0 | 0.0 | 9 | 4.5 |
| I always try to comply with the wishes of patients/relatives from an ethnic minority background. | 1 | 0.5 | 4 | 2.0 | 24 | 11.9 | 128 | 63.7 | 42 | 20.9 | 2 | 1.0 |
| In our department, we sometimes go to great lengths to accommodate the wishes of patients/relatives from an ethnic minority background. | 10 | 5.0 | 22 | 10.9 | 44 | 21.9 | 86 | 42.8 | 31 | 15.4 | 8 | 4.0 |
| The staff members often disagree about how far we should go to meet the wishes of patients/relatives from an ethnic minority background. | 27 | 13.4 | 62 | 30.8 | 55 | 27.4 | 34 | 16.9 | 1 | 0.5 | 22 | 10.9 |
| Balancing the needs of patients from an ethnic minority background and their relatives may occasionally conflict with the needs of Norwegian patients, such as the requirement for private rooms. | 5 | 2.5 | 28 | 13.9 | 55 | 27.4 | 76 | 37.8 | 6 | 3.0 | 31 | 15.4 |
| I feel uneasy about providing care for patients when there are many visitors in the room. | 30 | 14.9 | 51 | 25.4 | 46 | 22.9 | 48 | 23.9 | 16 | 8.0 | 10 | 5.0 |
| Working with ethnic minority patients requires more time and energy. | 4 | 2.0 | 19 | 9.5 | 79 | 39.3 | 80 | 39.8 | 12 | 6.0 | 7 | 3.5 |
| Ethnic minority patients have specific preferences regarding the gender of health personnel. | 10 | 5.0 | 33 | 16.4 | 59 | 29.4 | 52 | 25.9 | 15 | 7.5 | 32 | 15.9 |
| **Communication and collaboration with interpreters** | | | | | | | | |  | | | |
| It is difficult to communicate well with patients through an interpreter. | 8 | 4.0 | 50 | 24.9 | 47 | 23.4 | 83 | 41.3 | 11 | 5.5 | 2 | 1.0 |
| I am confident that the interpreter translates everything that is said. | 1 | 0.5 | 32 | 15.9 | 50 | 24.9 | 101 | 50.2 | 10 | 5.0 | 7 | 3.5 |
| I am confident that the interpreter will address any misunderstandings that arise during conversations. | 0 | 0.0 | 33 | 16.4 | 57 | 28.4 | 92 | 45.8 | 10 | 5.0 | 9 | 4.5 |
| I am aware of the appropriate channels for obtaining a professional interpreter. | 2 | 1.0 | 9 | 4.5 | 15 | 7.5 | 96 | 47.8 | 74 | 36.8 | 5 | 2.5 |
| **Death and dying** | | | | | | | | | | | | |
| I have sufficient knowledge of deathbed rituals when a patient from a minority background passes away and needs to be cared for. | 43 | 21.4 | 95 | 47.3 | 43 | 21.4 | 11 | 5.5 | 0 | 0.0 | 9 | 4.5 |
| I seek advice from relatives regarding care and rituals when a patient from a minority background passes away. | 0 | 0.0 | 6 | 3.0 | 40 | 19.9 | 88 | 43.8 | 27 | 13.4 | 40 | 19.9 |
| It is more difficult to relate to grieving relatives from an ethnic minority background. | 12 | 6.0 | 48 | 23.9 | 60 | 29.9 | 30 | 14.9 | 8 | 4.0 | 43 | 21.4 |
| I know who to contact in the event of the death of patients from an ethnic minority background. | 16 | 8.0 | 56 | 27.9 | 44 | 21.9 | 34 | 16.9 | 10 | 5.0 | 41 | 20.4 |
| **Culture, religion, and diet** | | | | | | | | | | | | |
| Patients from an ethnic minority background are offered food adapted to their faith and culture. | 4 | 2.0 | 11 | 5.5 | 33 | 16.4 | 109 | 54.2 | 29 | 14.4 | 15 | 7.5 |
| I have sufficient knowledge of religious dietary rules. | 5 | 2.5 | 45 | 22.4 | 70 | 34.8 | 60 | 29.9 | 11 | 5.5 | 10 | 5.0 |
| Muslim patients receive religiously adapted food (“halal food”), while patients with other religious backgrounds (Jews, Hindus, Buddhists, etc.) do not. | 6 | 3.0 | 39 | 19.4 | 30 | 14.9 | 36 | 17.9 | 8 | 4.0 | 82 | 40.8 |
| Most Muslim patients in my ward appreciate the halal food that is served. | 4 | 2.0 | 13 | 6.5 | 49 | 24.4 | 20 | 10.0 | 0 | 0.0 | 115 | 57.2 |

**Supplementary Table 2:** Proficiency in languages other than Norwegian

| **Language** | **Yes** | | **No** | |
| --- | --- | --- | --- | --- |
|  | **N** | **%** | **N** | **%** |
| English | 179 | 89.1 | 22 | 10.1 |
| German | 17 | 8.5 | 184 | 91.5 |
| Swedish | 10 | 5.0 | 191 | 95.0 |
| Danish | 9 | 4.5 | 192 | 95.5 |
| Spanish | 9 | 4.5 | 192 | 95.5 |
| Sami | 4 | 2.0 | 197 | 98.0 |
| Arabic | 4 | 2.0 | 197 | 98.0 |
| Russian | 4 | 2.0 | 197 | 98.0 |
| Finnish | 3 | 1.5 | 198 | 98.5 |
| Dutch | 3 | 1.5 | 198 | 98.5 |
| French | 3 | 1.5 | 198 | 98.5 |
| Polish | 3 | 1.5 | 198 | 98.5 |
| Ukrainian | 3 | 1.5 | 198 | 98.5 |
| Lithuanian | 2 | 1.0 | 199 | 99.0 |
| Thai | 2 | 1.0 | 199 | 99.0 |
| Somali | 2 | 1.0 | 199 | 99.0 |
| Tigrine | 2 | 1.0 | 199 | 99.0 |
| Portuguese | 1 | 0.5 | 200 | 99.5 |
| Latvian | 1 | 0.5 | 200 | 99.5 |
| Greek | 1 | 0.5 | 200 | 99.5 |
| Macedonian | 1 | 0.5 | 200 | 99.5 |
| Serbian | 1 | 0.5 | 200 | 99.5 |
| Vietnamese | 1 | 0.5 | 200 | 99.5 |
| Korean | 1 | 0.5 | 200 | 99.5 |
